# Sign of Pregnancy

**Published:** 1844-02-24

**Authors:** 


### SIGN OF PREGNANCY.

The surest token of pregnancy, says Dr. d'Outrepont, is a *dark-bluish hue* of the vaginal mucous membrane (*zunächst gelegenen scheidepartie*,) a symptom which is the more valuable inasmuch as it is not evanescent or confined to one period, but continues from the period of conception to that of labour. Dr. d'Outrepont, has not only met with this appearance uniformly in the human subject, but in different animals which he examined in in every period of pregnancy, and which he destroyed to ascertain that no fallacy attended the suspicion of of gestation.—*Nene Zeitschr. fur Geburtsh.*, xiv., 3.

